# Identification of *Echinococcus granulosus* Genotypes G1 and G3 by SNPs Genotyping Assays

**DOI:** 10.3390/pathogens10020125

**Published:** 2021-01-26

**Authors:** Piero Bonelli, Silvia Dei Giudici, Angela Peruzzu, Lorena Mura, Cinzia Santucciu, Caterina Maestrale, Giovanna Masala

**Affiliations:** 1 OIE Reference Laboratory for Echinococcosis, National Reference Center for Echinococcosis (CeNRE), IZS della Sardegna, 07100 Sassari, Italy; angela.peruzzu@izs-sardegna.it (A.P.); lorena.mura@live.it (L.M.); cinzia.santucciu@izs-sardegna.it (C.S.); giovanna.masala@izs-sardegna.it (G.M.); 2 Laboratory of Diagnostic Virology, Istituto Zooprofilattico della Sardegna (IZS), 07100 Sassari, Italy; silvia.deigiudici@izs-sardegna.it; 3 Laboratory of Anatomical Pathology, Histopathology, Animal Genetics, Istituto Zooprofilattico della Sardegna (IZS), 07100 Sassari, Italy; caterina.maestrale@izs-sardegna.it

**Keywords:** *Echinococcus granulosus sensu stricto*, genotypes G1 and G3, single nucleotide polymorphisms, real time PCR

## Abstract

*Echinococcus granulosus sensu lato* (*s.l.*) is the causative agent of cystic echinococcosis in animals and humans. Different *E. granulosus* *s.l.* genotypes exhibit great diversity in their life cycle, host selectivity and pathogenicity. For this reason, the study of genetic variation within *Echinococcus* species is of importance for their epidemiological implication. We employed two SNP genotyping technologies to distinguish G1 and G3 *E. granulosus sensu stricto (s.s.*). genotypes. The genotypes of DNA samples (n = 28) extracted from hydatid cysts of different animal species were identified by amplification and sequencing of a fragment of the mitochondrial nad5 gene. Two SYBR green and three TaqMan real time PCR assays were developed for targeting of three nad5 informative positions (SNP758, 1123, and 1380) known to be able to discriminate G1 from G3. Genotyping by SYBR Green PCR based on cycle threshold (Ct) with melting temperature (Tm) analysis and performed on SNP1123 and SNP1380 failed to identify one DNA sample. TaqMan assays for SNP758, 1123 and 1380 effectively confirmed genotype identification obtained by Sanger sequencing. Our results demonstrated that the combination of the three Taqman assays developed in this study represents a valuable and cost effective tool alternative to DNA sequencing for *E. granulosus s.s.* genotyping.

## 1. Introduction

The larval form of *Echinococcus granulosus sensu lato* (*s.l.*) is the etiological agent of cystic echinococcosis (CE). CE is a worldwide-distributed disease whose strong zoonotic character is cause of great concern for human health. Considering its consistent clinical and economic burden, the World Health Organization (WHO) included CE in a list of seven neglected zoonotic diseases requiring priority intervention [1]. The life cycle of *E. granulosus s.l.* is indirect and involves definitive and intermediate hosts. The adult parasite lives in the small intestine of the definitive hosts, mainly wild or domestic canids, responsible of eggs dispersion with the feces in the external environment. Intermediate hosts, usually ungulates, acquire infection by ingesting eggs that develop in the internal organs into the larval form (metacestode). The cycle is completed when the definitive hosts feed on infected organs of intermediate hosts. Accidentally humans can ingest eggs and act as aberrant hosts.

Molecular phylogenetic reconstruction based on mitochondrial (mt) genes had suggested the taxonomic subdivision of *E. granulosus s.l.* into five species: E. *granulosus sensu stricto* (*s.s.*), *Echinococcus ortleppi*, *Echinococcus equinus*, *Echinococcus canadensis* and *Echinococcus felidis* [2,3]. *E. granulosus s.s.* is widespread globally, reaching high prevalence rates in some specific endemic areas as South America, Mediterranean countries and Asia; it is generally maintained by a domestic dog-sheep cycle [4] and considered to be responsible for the vast majority of human and livestock CE cases worldwide [5]. Three different genotypes were firstly assigned to *E. granulosus s.s.* [6]: G1 genotype (formerly also known as the sheep strain), G2 genotype (Tasmanian sheep strain), and G3 genotype (buffalo strain) [7]. However, recent studies performed on mitochondrial and nuclear genetic markers considered G2 as a variant of G3 rather than a distinct genotype [8]. Originally *Echinococcus* genotyping relied on the analysis of a short fragment (366 bp) of the mt cox1 gene [9] that led to the identification of the *E. granulosus s.s.* G1–G3 genotypes. Over the past years, many studies on genetic diversity of *E. granulosus* have been based on the mtDNA sequences of the cox1 gene [3,10,11,12,13,14], but it was not until recently [15,16] that the analysis of larger portions of mt genome evidenced how the cox1 gene could not clearly differentiate *E. granulosus s.s.* genotypes. A study published by Kinkar et al. [17], based on a large dataset of near complete mitogenomes, evidenced instead, how a relatively short fragment of the nad5 gene could provide a consistent identification of *E. granulosus s.s.* G1 and G3 genotypes. Six informative positions were found, with three single nucleotide polymorphisms (SNPs) being able to reliably distinguish G1 from G3 genotype. 

SNPs are point mutations, caused by a single base substitution, considered one of the most common form of genome variation [18]. Since the past years, the usage of molecular markers in biology has been largely applied from evolutionary studies to diagnostic purposes [19,20]. Particularly, SNPs are powerful tool for genetic diversity analysis able to establish differences between individuals, populations and species [21,22,23]. In this study we proposed two SNP genotyping methods, for the distinction of *E. granulosus s.s.* G1 and G3 genotypes, employing SYBR Green and TaqMan real-time PCR chemistries. 

## 2. Results

### 2.1. Genotyping by DNA Sequencing

A fragment of the mt nad5 gene was successfully amplified and sequenced from 28 hydatid cysts samples collected in this study. DNA consensus sequences of 670 bp were obtained by trimming low quality chromatogram data. *E. granulosus s.s* genotypes were identified by analyzing three SNPs at position 758, 1123, and 1380 according to GenBank reference sequence AB786664 as reported by Kinkar et al. [17]. DNA isolates characterized by the presence of G at the three informative positions were considered G1; whereas those with C at position 758, and with A at position 1123 and 1380, were assigned to G3 genotype. Out of 28 sequences, 16 were identified as G1 and 12 as G3 genotype (Table A1). 

The haplotype sequences analyzed in this study were deposited in GenBank under the following accession number MT993962-MT993973 (Table A1). 

### 2.2. Genotyping by SYBR Green PCR 

**Figure 1:**
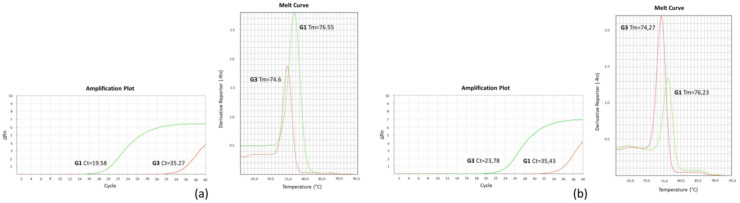
A representative analysis of G1 and G3 genotype samples by SYBR green PCR: (**a**) the sample displaying an amplicon with a Ct < 30 and the higher Tm value was considered G1 (**b**) the sample displaying an amplicon with a Ct < 30 and the lower Tm value was considered G3.

We aimed to develop a SYBR green PCR assay to target the three SNPs at position 758, 1123, and 1380 in order to discriminate G1 and G3 *E. granulosus s.s*. genotypes by combining assessment of cycle threshold (Ct) with melting temperature (Tm) analysis [24]. To this purpose we tried to design three primer sets consisting of two forward primers, each specific for each SNP and incorporating GC tails of different lengths, and a common reverse primer. NetPrimer found the primer sets for both SNP 1123 and 1380 but did not return any effective option for SNP758. The forward primers specific for G1 were provided with a longer GC tail respect to the forward primers designed for G3, so that two PCR products of different size and melting temperature were amplified (Table 1). Each primer set produced two amplicons per sample. Only amplicons with a Ct value less than 30 were considered for the analysis. G1 genotype was assigned to those samples characterized by the presence of an amplicon with a Ct < 30 and the higher value of Tm (Figure 1a). On the contrary, G3 genotype was assigned to those samples showing an amplicon with Ct < 30 and the lower value of Tm (Figure 1b). In Table 2 the Ct and Tm mean values observed analyzing G1 and G3 samples for SNP 1123 and SNP 1380 are reported. The Tm differences (ΔTm) between amplicons related to G1 and G3 genotypes were of 1.82 °C in SNP 1123 and 2.22 °C in SNP 1380. 

The genotype identification of all the samples analyzed by SYBR Green PCR was in agreement with DNA sequencing except for one sample (sample 21). This sample was identified by SNP1123-based assay as G3 confirming what obtained by DNA sequencing, but the results from SNP1380-based assay were inconclusive (Table A1). 

### 2.3. Genotyping by TaqMan PCR

**Figure 2:**
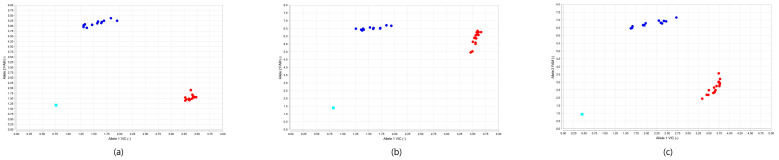
A representative analysis of G1 and G3 genotype samples by TaqMan PCR. Data plots were calculated by TaqMan Genotyper Software. FAM fluorophore (y-axis) is associated with the G1 probe, VIC fluorophore (x-axis) labels the G3 probe. G1 samples are represented by red dots, G3 samples by blue dots, NTC by grey squares: (**a**) Genotype discrimination plot for SNP758. (**b**) Genotype discrimination plot for SNP1123. (**c**) Genotype discrimination plot for SNP1380. NTC (No Template Control), FAM (5(6)-carboxyfluorescein), VIC (2′-chloro-7′phenyl-1,4-dichloro-6-carboxy-fluorescein).

A single well TaqMan PCR method (Applied Biosystems, Foster City, CA, USA) for SNP 758, 1123, and 1380 consisting of a primers pairs and two probes, each specific for G1 and G3 genotype (Table 3), was performed to analyze the DNA samples collected in this study. Data were examined using a software algorithm which graphically represents each sample as an independent data point on a genotype discrimination plot (Figure 2). All samples belonging to the same genotype will cluster together on the plot and the relative position of the clusters will determine their automatic genotype call.

The genotype of the analyzed samples were identified by the three Real Time PCR assays and the results were in perfect agreement with those obtained by DNA sequencing (Table A1). As showed in Figure 2, the data points in the genotype discrimination plots appeared to be appropriately structured in separate and compact clusters allowing an effective discrimination of all the samples analyzed. However, data obtained by SNP758-based assay generated better genotype calls enabling the *E. granulosus s.s.* genotypes identification without the need of any improvement to the automatic classification proposed by the software algorithm (Figure 2a). In order to optimize the results of SNP1123 and SNP1380 assays (Figure 2b,c), obtained respectively for the G1 cluster and one G3 sample, the automatic calls were reviewed and the genotype call manually assigned on the basis of the quality value of each data point and its position on the discrimination plot.

## 3. Discussion

In this study we aimed to identify an efficient SNP-based molecular assay able to distinguish *E. granulosus s.s.* G1 and G3 genotype. Twenty-eight hydatid cysts were sampled and molecularly characterized by Sanger sequencing. Two SYBR green and three TaqMan real time PCR assays for SNP genotyping were tested for their ability to discriminate G1 from G3 genotype.

DNA was extracted from parasite material collected from infected organs of different intermediate hosts (Table A1) and a fragment of the mitochondrial nad5 gene was amplified and sequenced for *E. granulosus s.s.* genotype identification [17]. Data from analysis of SNP758, 1123, and 1380, identifying 16 G1 and 12 G3 nucleotide sequences (Table A1), were used as reference to evaluate the results of the real time PCR assays designed in this study.

In the past years, several methods for SNP analysis using real-time PCR were proposed as alternative technologies to DNA sequencing [19,25,26]. Among these techniques the Tm-shift analysis [24,27,28,29] and the TaqMan system [30,31,32] have been widely used for allelic and genotypic discrimination in a variety of biomedical disciplines including veterinary science. In our investigation, we intended to develop three SYBR green PCR assays to detect nad5 SNP 758, 1123, and 1380, but NetPrimer was not able to design reliable primers sets for SNP 758. Instead, SNP1123 and SNP1380 primer sets were found and used to test DNA samples, with the resulting data examined combining Ct assessment with Tm analysis. In spite of the greater sizes of SNP1123 amplicons compared to those of SNP1380, lower Tm values were recorded for SNP1123, possibly due to their corresponding GC content (Table 1). The differences between Tm of G1 and G3 PCR products (ΔTm) did allow a proper discrimination in both assays (ΔTm SNP1123 = 1.82 °C, ΔTm SNP1380 = 2.2 °C). The SNP1380-based assay failed to discriminate a G3 sample (sample 21) that was correctly identified by targeting the SNP1123. Sample 21 showed Ct values lower than 30 for both amplicons (G1_Ct = 22.18 G3_Ct = 18.00) resembling the pattern of a coinfection with both genotypes, that was not evidenced by the other genotyping methods used in this study. Furthermore, the sample 21 belongs to the same haplotype of other sequences that were appropriately identified by SNP1380 assay. The quantity and quality of DNA are critical to the success of the target amplification; thus, the possibility that the assay could really identify coinfection is to be confirmed with a larger samples set. As shown in Table A1, the rest of the samples were properly identified yielding identical results as the DNA sequencing by using both the SYBR green PCR assays.

The TaqMan system is one of the first method proposed for SNP genotyping, its use being widely distributed for the high-throughput and sensitivity features [32]. In this study, we tested three TaqMan PCR assays that proved to correctly identify *E. granulosus s.s.* genotypes. Data analysis was performed by the autocalling option of the Taqman Genotyper Software using an algorithm able to assign a genotype to each sample. The software results were reviewed and when necessary manually revised on the basis of the quality value of the data points and its position on the discrimination plot. The SNP758 TaqMan PCR showed to accurately place samples in well separated and compact clusters (Figure 2a). SNP1123 assay assigned to the entire G1 cluster a position in the data plot that was interpreted as a mixed genotype (G1/G3) by the autocalling method of the software. Because of the high intensity signal detected for both G1 and G3 probes, the corresponding cluster showed intermediate features occupying a position equidistant of the data plot’s axes that the algorithm read as the coexistence of two genotypes. This may be possibly explained by the same binding affinity of the two probes that specifically occurred when targeting SNP1123 in G1 samples. To improve effectiveness of the genotype assignment a manual call was performed on those data points that clearly belonged to the G1-cluster (Figure 2b). The genotype discrimination plots calculated for the SNP1380 assay (Figure 2c) showed a more dispersed G3-cluster compared to what observed for SNP758 and SNP1123 assays. All the samples were correctly genotyped automatically by the software except for one undetermined sample (sample 13) positioned at the top edge of the G1 cluster. Still, for its proximity to the other data points it was considered as part of the G1 cluster and consequently identified. 

Although the SYBR green assays designed in this study demonstrated the ability to correctly identify almost all the samples analyzed (Table A1), we would be inclined to suggest the use of the TaqMan method for *E. granulosus s.s* genotypes discrimination. Because of the high genetic diversity of *E. granulosus s.l.* it is likely that the three SNPs selected for this study might not be completely fixed for G1 and G3 genotypes. Even though the three assays, especially SNP 758, seemed able to separately distinguish G1 from G3, we cannot exclude the existence of rare genetic variants characterized by the absence of the diagnostic polymorphism examined in this study. For this reason, as already pointed out by other authors [17,33], the targeting of the single SNP cannot be sufficient to reliably distinguish G1 from G3 but the use of all three SNPs is highly recommended. The combination of the three Taqman PCR-based assays can be considered a valuable and cost effective tool alternative to DNA sequencing for *E. granulosus s.s.* genotyping. Compared to nad5 sequence analysis, requiring different operational phases and relevant technical competencies, TaqMan PCR can be considered a faster tool with the advantage of a simple execution and interpretation of the results. Considering the pronounced variability exhibited by the different *E. granulosus* genotypes in terms of epidemiology, life cycle, host selectivity, and pathogenicity, we believe that the development of a new and easy diagnostic tool may be of potential significance for CE control strategy.

## 4. Materials and Methods 

### 4.1. Samples Collection

Parasite material from hydatid cysts (n = 28) were collected from intermediate hosts of different animal species in Sardinia (Italy). Genomic DNA was extracted from protoscoleces or germinal layers using the Dneasy Blood and Tissue Kit (Qiagen, Hilden, Germany). DNA concentration and quality were measured by spectrophotometry (Implen, Munich, Germany), the samples were stored at −80 °C before analyses.

### 4.2. Sequencing of the Mitochondrial nad5 Genes 

A fragment of the mitochondrial nad5 gene was amplified as already described [17]. Sanger sequencing was performed on both strands with the same primers used for the amplification on an ABI-PRISM 3500 Genetic Analyzer (Applied Biosystems, Foster City, CA, USA) with dRhodamine Terminator Cycle Sequencing kit (Applied Biosystems, Foster City, California, USA). The consensus sequences were assembled and edited in the BioEdit software v7.0.0 [34]. Genotypes of *E. granulosus s.s.* were identified based on three nucleotide positions (758, 1123, 1380 according to GenBank reference sequence AB786664) within nad5 gene fragment as reported by Kinkar [17]. 

### 4.3. SNP Genotyping

SNP genotyping based on nad5 mitochondrial gene for distinction of *E. granulosus s.s.* G1 and G3 genotypes was performed using two different Real Time PCR chemistries. 

SYBR Green PCR with SNP specific primers and TaqMan PCR with SNP specific probes assays were designed at the informative positions 758, 1123, and 1380. The position 758 is characterized by the presence of G or C in G1 and G3 genotypes respectively, instead the positions 1123 and 1380 were characterized by the presence of G or A correspondingly. In very rare cases, nucleotide C was found in place of nucleotide G in G1 samples [17]. 

#### 4.3.1. SYBR Green PCR with SNP Specific Primers

The method, already described by other authors [24,29], was carried out using two forward primers with varying nucleotide at its 3’ end, specific to each SNP, and a common reverse primer. A mismatched base before 3’ end of the forward primers was incorporated to increase the specificity of the PCR. Short 6-bp and long 14-bp GC tails were added at the 5’end of each forward primers to produce amplicons of different lengths and melting temperature. Primers were designed using the online software, NetPrimer (Premier Biosoft, San Francisco, CA, USA). The sequence and characteristics of primers used are reported in Table 1. No primers were found by NetPrimer for SNP 758. Two reaction mixtures were prepared for each SNP-based assay differing for the presence of the forward primer specifically designed for G1 and G3 genotypes DNA sequence. PCR amplification was performed in a volume of 20 µL in two distinct wells containing, respectively 0.1 µM of forward primer and 0.1 µM common reverse primer for each SNP, 1x Power SYBR Green master mix (Thermo Fisher, Waltham, MA, USA) and 50 ng of DNA template. Real time PCR and Melting Curve Analysis were carried out using 7500 Fast Real Time PCR System (Thermo Fisher, Waltham, MA, USA) with the following protocol: initial denaturation 95°C for 10 min, followed by 45 cycles of 95 °C for 15 s, 54 °C for 30 s, 60 °C for 1 min. The amplicon Tm was obtained collecting fluorescence data from 60 °C to 95 °C following the default instrument conditions. G1 and G3 genotypes were distinguished by their Ct and Tm values as previously reported [24]. Briefly, sample results were examined on the PCR products characteristics of two wells, one for each forward primers of different length. G1 specific forward primer produced a longer amplicon than G3 specific primer. Amplicons with higher Tm were considered G1, whereas samples with lower Tm values were identified as G3. Only the amplifications with Ct < 30 were considered for the analysis. This cycle threshold value was chosen on the basis of the results obtained in repeated trials with all the samples analyzed in this study and confirmed by DNA sequencing analysis. In Figure 1 a representative SYBR green PCR analysis of G1 (Figure 1a) and G3 genotype (Figure 1b) samples is illustrated.

#### 4.3.2. TaqMan PCR with SNP Specific Probes 

Three single well custom-made assays for SNP 758, 1123, and 1380, consisting of a primers pairs and two probes each recognizing G1 or G3 genotype, were created by Thermo Fisher (Waltham, MA, USA). The sequence and characteristics of primers and probes used in this study are reported in Table 3. PCR amplification was performed in a volume of 20 µL containing 0.9 µM of each primer, 0.2 µM of each probe, 1x PCR master mix (Solis Biodyne, Tartu, Estonia) and 10 ng of template DNA. Real time PCR was carried out using 7500 Fast Real Time PCR System (Thermo Fisher, Waltham, MA, USA) with the following protocol: initial denaturation 95 °C for 10 min, followed by 40 cycles of 95 °C for 15 s, 60 °C for 1 min. Data were analyzed by TaqMan Genotyper Software (Thermo Fisher, Waltham, MA, USA) and reported in a genotype discrimination plot where the samples sharing the same single-base mutation constituted a separate data points cluster. The software algorithm determines the probability that each data point belongs to a given category by measuring its signal intensity, as the distance of the data point from the no template control (NTC), and the angle formed by the data point with respect to the x-axis and NTC as the origin. Each genotype call probability is expressed by a numeric quality value which is function of the cluster distance from NTC, cluster separation and cluster dispersion [35]. The autocalling option of the TaqMan Genotyper Software, allowing to perform a genotype assignment to each sample was used. In order to improve the accuracy of the automated genotype calling, the results were analyzed by a skilled operator and, if necessary, the autocalled genotypes reviewed by manual calling.

## Figures and Tables

**Table 1:** SYBR Green PCR: specific primers designed for SNP 1123 and 1380. The underlined nucleotides marked in bold at the 5’end represent the GC tails.

SNP	Primer	Sequence	Product Length (bp)	Product GC Content (%)
1123	SNP_1123_G1 Fw SNP_1123_G3 Fw SNP_1123 Rv	5’-**GCGGGCAGGGCGGC**CGTTATGACTATTTGTTACATTTgG-3’ 5’-**GCGGGC**CGTTATGACTATTTGTTACATTTgA-3’ 5’-ACAAAGCCACAATCTTCTTC-3’	141 133	41.10 37.68
1380	SNP_1380_G1 Fw SNP_1380_G3 Fw SNP_1380 Rv	5’- **GCGGGCAGGGCGGC**GGGTGGTTCACAGGCTAaG -3’ 5’- **GCGGGC**GGGTGGTTCACAGGCTAaA -3’ 5’- AACAACCCAAACAAATTCC -3’	86 78	53.85 49.40

**Table 2:** Ct and Tm values of the amplicons containing single nucleotide polymorphisms (SNPs) diagnostic for G1 and G3 genotypes obtained by SNP1123 and SNP1380 SYBR green PCR.

	Genotype G1		Genotype G3	
	Ct (Mean ± SD)	Tm °C (Mean ± SD)	Ct (Mean ± SD)	Tm °C (Mean ± SD)
SNP1123	21.52 ± 3.72	76.51 ± 0.22	26.42 ± 2.87	74.69 ± 0.30
SNP1380	21.63 ± 3.12	79.09 ± 0.29	24.10 ± 2.95	76.87 ± 0.23

**Table 3:** TaqMan real-time PCR assays designed for SNP 758, 1123, and 1380.

SNP	Primer	Sequence
758	SNP_758_F SNP_758_R Probe_G1 Probe_G3	5’-GGTTTATGTTGTTGAAGTTGATTGTTTTGT-3’ 5’-AAAACCTAACAACACCTAAATACTCTCAAAGAA-3’ VIC-5’- TGTTGGTATGTAGTGGTGAT-3’ FAM-5’- TGTTGGTATGTACTGGTGAT-3’
1123	SNP_1123_F SNP_1123_R Probe_G1 Probe_G3	5’-CTGGTGTTTGGTTTGTTATGCGTTA-3’ 5’-CCAGTAATAAAAACCGTCAACAAAAGCA-3’ VIC-5’-CGACCTACCAAAATG-3’ FAM-5’- CCGACCTACTAAAATG-3’
1380	SNP_1380_F SNP_1380_R Probe_G1 Probe_G3	5’-GTGATGTGATGAGCGGTAGGG-3’ 5’-CACGACCCATACAAAACAGACCTAT-3’ VIC-5’- CAGGCTAGGAATTGT-3’ FAM-5’-CAGGCTAGAAATTGT-3’

## Data Availability

The dataset produced in this study is openly available in GenBank (see Table A1 for accession number).

## Appendix A

In Table A1 the genotype identification of the 28 samples collected from different animal species for this study are reported. The results from Sanger sequencing were used as reference to be compared with data of SNP genotyping performed in real time PCR. Two SYBR green assays targeting SNP1123 and SNP1380 and three TaqMan PCR assays targeting SNP 758, SNP 1123 and SNP1380 were developed.

**Table A1:** *Echinococcus granulosus s.s.* isolates identified by Sanger sequencing and by SNP genotyping in real time PCR using SYBR green and TaqMan chemistries.

ID	Host	Isolate	Genotyping Method						Haplotype	Accession Number
			Sanger sequencing	SYBR green PCR		TaqMan PCR				
			nad5 (670 bp)	SNP 1123	SNP1380	SNP758	SNP 1123	SNP1380		
1	ovine	16762	G1	G1	G1	G1	G1	G1	nd5SAR1	MT993962
2	ovine	16767	G1	G1	G1	G1	G1	G1	nd5SAR2	MT993963
3	caprine	30487	G1	G1	G1	G1	G1	G1	nd5SAR3	MT993964
4	ovine	40243	G1	G1	G1	G1	G1	G1	nd5SAR4	MT993965
5	bovine	50508	G1	G1	G1	G1	G1	G1	nd5SAR1	MT993962
6	bovine	51056	G1	G1	G1	G1	G1	G1	nd5SAR7	MT993968
7	bovine	53965	G1	G1	G1	G1	G1	G1	nd5SAR1	MT993962
8	bovine	60008	G1	G1	G1	G1	G1	G1	nd5SAR8	MT993969
9	ovine	70802	G1	G1	G1	G1	G1	G1	nd5SAR5	MT993966
10	bovine	74248	G1	G1	G1	G1	G1	G1	nd5SAR4	MT993965
11	bovine	76558	G1	G1	G1	G1	G1	G1	nd5SAR6	MT993967
12	ovine	76728	G1	G1	G1	G1	G1	G1	nd5SAR1	MT993962
13	ovine	78417	G1	G1	G1	G1	G1	G1	nd5SAR1	MT993962
14	bovine	78434	G1	G1	G1	G1	G1	G1	nd5SAR1	MT993962
15	bovine	82760	G1	G1	G1	G1	G1	G1	nd5SAR1	MT993962
16	human	82942	G1	G1	G1	G1	G1	G1	nd5SAR9	MT993970
17	ovine	35378	G3	G3	G3	G3	G3	G3	ndSAR11	MT993972
18	bovine	42450	G3	G3	G3	G3	G3	G3	nd5SAR9	MT993970
19	bovine	50501	G3	G3	G3	G3	G3	G3	nd5SAR4	MT993965
20	bovine	53437	G3	G3	G3	G3	G3	G3	nd5SAR9	MT993970
21	ovine	57455	G3	G3	G1/G3	G3	G3	G3	nd5SAR9	MT993970
22	ovine	68923	G3	G3	G3	G3	G3	G3	nd5SAR9	MT993970
23	ovine	70804	G3	G3	G3	G3	G3	G3	nd5SAR9	MT993970
24	human	75588	G3	G3	G3	G3	G3	G3	nd5SAR9	MT993970
25	ovine	77580	G3	G3	G3	G3	G3	G3	nd5SAR9	MT993970
26	human	84912	G3	G3	G3	G3	G3	G3	nd5SAR9	MT993970
27	caprine	95256	G3	G3	G3	G3	G3	G3	ndSAR10	MT993971
28	human	103212	G3	G3	G3	G3	G3	G3	ndSAR12	MT993973
